# Factors Associated With the Intention to Receive the COVID-19 Vaccine: Cross-sectional National Study

**DOI:** 10.2196/37203

**Published:** 2022-11-14

**Authors:** Monica L Kasting, Jonathan T Macy, Shaun J Grannis, Ashley J Wiensch, Juan M Lavista Ferres, Brian E Dixon

**Affiliations:** 1 Department of Public Health Purdue University West Lafayette, IN United States; 2 Cancer Prevention and Control Program Simon Comprehensive Cancer Center Indiana University Indianapolis, IN United States; 3 Department of Applied Health Science School of Public Health Indiana University Bloomington, IN United States; 4 Department of Family Medicine School of Medicine Indiana University Indianapolis, IN United States; 5 Center for Biomedical Informatics Regenstrief Institute Indianapolis, IN United States; 6 AI for Good Research Lab Microsoft Corporation Redmond, WA United States; 7 Richard M Fairbanks School of Public Health Indiana University Indianapolis, IN United States; 8 Center for Health Information and Communication Health Services Research & Development Service Richard L Roudebush VA Medical Center, Veterans Health Administration Indianapolis, IN United States

**Keywords:** SARS-CoV-2, COVID-19 vaccines, vaccination intention, vaccine hesitancy, Health Belief Model, reasoned action approach, COVID-19, vaccination, public health, online survey, health intervention, logistic regression, demographic

## Abstract

**Background:**

The COVID-19 pandemic is an unprecedented public health crisis, and vaccines are the most effective means of preventing severe consequences of this disease. Hesitancy regarding vaccines persists among adults in the United States, despite overwhelming scientific evidence of safety and efficacy.

**Objective:**

The purpose of this study was to use the Health Belief Model (HBM) and reasoned action approach (RAA) to examine COVID-19 vaccine hesitancy by comparing those who had already received 1 vaccine to those who had received none.

**Methods:**

This study examined demographic and theory-based factors associated with vaccine uptake and intention among 1643 adults in the United States who completed an online survey during February and March 2021. Survey items included demographic variables (eg, age, sex, political ideology), attitudes, and health belief variables (eg, perceived self-efficacy, perceived susceptibility). Hierarchical logistic regression analyses were used for vaccine uptake/intent. The first model included demographic variables. The second model added theory-based factors to examine the association of health beliefs and vaccine uptake above and beyond the associations explained by demographic characteristics alone.

**Results:**

The majority of participants were male (n=974, 59.3%), White (n=1347, 82.0%), and non-Hispanic (n=1518, 92.4%) and reported they had already received a COVID-19 vaccine or definitely would when it was available to them (n=1306, 79.5%). Demographic variables significantly associated with vaccine uptake/intent included age (adjusted odds ratio [AOR] 1.05, 95% CI 1.04-1.06), other race (AOR 0.47, 95% CI 0.27-0.83 vs White), and political ideology (AOR 15.77, 95% CI 7.03-35.35 very liberal vs very conservative). The theory-based factors most strongly associated with uptake/intention were attitudes (AOR 3.72, 95% CI 2.42-5.73), self-efficacy (AOR 1.75, 95% CI 1.34-2.29), and concerns about side effects (AOR 0.59, 95% CI 0.46-0.76). Although race and political ideology were significant in the model of demographic characteristics, they were not significant when controlling for attitudes and beliefs.

**Conclusions:**

Vaccination represents one of the best tools to combat the COVID-19 pandemic, as well as other possible pandemics in the future. This study showed that older age, attitudes, injunctive norms, descriptive norms, and self-efficacy are positively associated with vaccine uptake and intent, whereas perceived side effects and lack of trust in the vaccine are associated with lower uptake and intent. Race and political ideology were not significant predictors when attitudes and beliefs were considered. Before vaccine hesitancy can be addressed, researchers and clinicians must understand the basis of vaccine hesitancy and which populations may show higher hesitancy to the vaccination so that interventions can be adequately targeted.

## Introduction

The COVID-19 pandemic, caused by the novel SARS-CoV-2 virus [1], represents an unprecedented public health crisis. On March 11, 2020, the World Health Organization officially declared COVID-19 a pandemic [1]. In less than 2 years, over 67 million cases and 850,000 deaths from COVID-19 occurred in the United States alone [2]. In December 2020, the US Food and Drug Administration (FDA) granted emergency use authorization for the first vaccine to protect against COVID-19. By April 2021, the FDA has issued emergency use authorization for vaccines by 3 different companies: Pfizer-BioNTech, Moderna, and Johnson & Johnson [3]. As of August 23, 2021, the FDA had granted full approval to the Pfizer-BioNTech vaccine [4]. In addition to data provided by the manufacturers to the FDA, multiple independent research studies demonstrate the vaccines are safe, effective, and widely available for individuals 5 years and older in the United States [5-7].

Hesitancy regarding COVID-19 vaccines persists among adults in the United States [8-12], despite overwhelming scientific evidence of their safety and efficacy. Vaccine hesitancy refers to delay in acceptance or refusal of vaccination, despite the availability of the vaccine and vaccine services [13]. This belief results in lower uptake of prophylactic vaccines and unnecessary morbidity and mortality from vaccine-preventable diseases [11,14,15]. Before vaccine hesitancy can be addressed through population-level intervention, researchers must better understand the basis of vaccine hesitancy and which populations may show higher hesitancy to the COVID-19 vaccine so that interventions can be adequately targeted.

Some of the strongest predictors of vaccine hesitancy and vaccine uptake are attitudes and beliefs derived from the Health Belief Model (HBM) and the reasoned action approach (RAA). Specifically, the HBM proposes that people will take action to prevent a disease if they believe that (1) they are susceptible, (2) the consequences are serious, (3) they can reduce susceptibility or severity through some action, (4) the benefits of taking action outweigh the barriers, and (5) they can engage in a specific behavior (self-efficacy) [16-19]. Previous research guided by this model shows vaccine intent and uptake across multiple diseases are associated with higher perceived benefits, lower perceived barriers, higher perceived severity of the disease, and higher perceived susceptibility/threat of disease [20-23]. However, because the current vaccines against COVID-19 were only approved for emergency use in December 2020, it is unknown whether these health beliefs will translate to how individuals perceive the new vaccine.

In addition to the HBM, this study is also informed by the RAA, which is the newest formulation of the theory of planned behavior and the theory of reasoned action [24]. The RAA extends the theory of planned behavior by differentiating between the attitude, subjective norms, and perceived behavioral control constructs that were integral in the original model [24]. RAA constructs, including experiential attitude, instrumental attitude, and injunctive norm, are significantly associated with the intent to engage in health behaviors [25]. Specifically, research shows that RAA constructs, in particular attitudes toward vaccination and perceived norms, are significantly associated with vaccine intent [26,27].

Therefore, the purpose of this study was to use the HBM and the RAA to examine COVID-19 vaccine hesitancy by exploring vaccine uptake and intent among a national convenience sample of adults in the United States. Specifically, we examined those who already received at least 1 dose of the COVID-19 vaccine or reported a strong intent to be vaccinated compared to those who did not report a strong likelihood of getting vaccinated, as well as demographic, attitudinal, and health belief variables associated with vaccination. Examining factors associated with vaccine uptake and intent provides valuable insight to inform future interventions to combat vaccine hesitancy, not only during the ongoing COVID-19 pandemic, but also during possible future pandemics.

## Methods

### Participants and Recruitment

**Figure 1:**
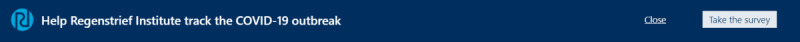
Study recruitment banner advertisement on Microsoft News.

We used recruitment methods developed during a pilot study by our team and previously published elsewhere [28]. Briefly, we partnered with Microsoft News to recruit participants to complete a 1-time online survey between February 25 and March 22, 2021. The survey questionnaire was developed for this study. The Microsoft News team created a banner advertisement, shown in Figure 1, which appeared across the top of a news page that a user was viewing. Microsoft News consumers with US browser settings were shown the survey twice in total if they did not click on it and never again after they clicked the link, regardless of whether they completed the survey. The link to the survey was additionally placed in an informational section of the Bing COVID-19 Tracker. Interested participants clicked on the banner and were directed to a survey developed using Qualtrics, a cloud-based survey tool licensed by Indiana University. Eligibility criteria included age 18 years or older, residing in the United States, and able to read English. The survey consisted of 35 individual questions and took approximately 5-10 minutes to complete, and participants were not provided with an incentive.

### Ethical Considerations

The study was given exempt status by the Indiana University Institutional Review Board. Because this was exempt research and no identifiable data were collected, this study received a waiver and did not collect written informed consent.

### Measures

The primary outcome for this study was vaccine uptake or intent (among the unvaccinated). Vaccine uptake was measured with the question “Have you received at least one dose of any COVID-19 vaccine?” Response options included “Yes, I have received one dose of a vaccine,” “Yes, I have received two doses of a vaccine,” or “No, I have not received a dose of any vaccine.” The people who had not received any doses of a COVID-19 vaccine were asked their vaccine intent with the question “If the vaccines were available where you live and offered to you at no cost, which of the following statements best describes your intention to get either of the vaccines?” Responses were scored on a 4-point Likert scale from “I would definitely get one of the vaccines” to “I would definitely not get either of these vaccines.” Responses to these 2 questions were dichotomized such that the sample was divided into those who had already received at least 1 dose or indicated they definitely would get the vaccine (vaccinated/intenders) and those who had not received the vaccine and indicated they did not intend to get vaccinated (unvaccinated/nonintenders).

Covariates fell into 2 categories: demographic characteristics and theory-based attitudes and beliefs. Demographic characteristics included age, gender (female, male, nonbinary, no response), race (White, Asian, Black/African American, or other), ethnicity (yes/no Latinx ethnicity), and political ideology (on a 5-point scale from very conservative to very liberal).

Theory-based attitudes and belief variables were measured on a 5-point Likert scale from strongly agree to strongly disagree. Attitudes about getting the vaccine were assessed with the statement “Getting vaccinated is a good thing to do.” To assess injunctive norms, we used the statement “Most people important to me think I should get vaccinated.” The descriptive norms construct was measured with the statement “Most people like me will get vaccinated.” To assess self-efficacy, participants responded to the statement “I am confident that I can get vaccinated.” To assess perceived susceptibility to COVID-19, participants responded to the statement “I am worried about the likelihood of getting COVID-19 in the near future.” We examined 3 separate barriers to vaccination: side effects (“The side effects of getting vaccinated interfere with my usual activities”), fear of needles (“I am scared of needles”), and trust in the vaccine (“I do not trust the vaccine”). All 3 items used the same 5-point Likert scale from strongly agree to strongly disagree and were analyzed as separate items.

### Data Analysis

First, we described the study sample using n (%) or means and SDs. We then compared the vaccinated/intenders group (already received at least 1 vaccine dose or reported they definitely will get vaccinated) and the unvaccinated/nonintenders group using chi-square or *t* tests, as appropriate. We then conducted a hierarchical logistic regression analysis. We first added the demographic covariates age, gender, race, ethnicity, and political ideology. We next added the theory-based factors to test their unique contributions independent from demographic influences. Analyses were conducted using IBM SPSS Statistics version 28.

## Results

### Participant Details

A total of 1643 people participated in the survey between February 25 and March 22, 2021, and reported their vaccine status. The sample was 59.3% (n=974) male, 82.0% (n=1347) White, and 92.4% (n=1518) non-Hispanic, and the mean age was 59.4 (SD 14.6, range 18-105) years. There was representation in the sample from all 50 states as well as Washington, DC, and Puerto Rico. For political ideology, 5.5% (n=90) of the participants reported being very conservative, 16.3% (n=268) were conservative, 37.3% (n=613) were moderate, 19.2% (n=316) were liberal, 9.4% (n=154) were very liberal, and 12.3% (n=202) did not respond. Overall, the majority (n=920, 56.0%) were unvaccinated, with 345 (21.0%) receiving 1 dose of any vaccine and 378 (23.0%) receiving 2 doses. Of the unvaccinated, 583 (63.4%) reported they definitely will get the vaccine, 104 (11.3%) reported they probably will get the vaccine, 65 (7.1%) reported they probably will not get the vaccine, and 168 (18.3%) reported they definitely will not get the vaccine. Therefore, for the purposes of this analysis, the majority (n=1306, 79.5%) reported already being vaccinated or said they definitely will get vaccinated when it is available to them. The mean age for the vaccine-hesitant group was slightly less compared to the vaccinated group (53.4 vs 60.9 years, *P*<.001). Vaccine uptake/intent differed by political ideology, with 37.4% (n=126) of the vaccine-hesitant group reporting being either very conservative or conservative. In contrast, only 17.7% (n=232) of the vaccinated/intenders group reported being very conservative (n=50, 21.6%) or conservative (n=182, 78.4%; *P*<.001). For a sample description and bivariate comparisons of the 2 groups, see Table 1.

**Table 1:** Sample characteristics by vaccine hesitancy.

Characteristics		Total (N=1643)	Vaccinated/intenders (n=1306, 79.5%)	Unvaccinated/nonintenders (n=337, 20.5%)
Age (years), mean (SD); *t* (*df*)=7.81 (1642), *P*<.001		59.4 (14.6)	60.9 (13.7)	53.4 (16.2)
**Gender, n (%); *χ*^2^=40.57, *P*<.001**				
	Female	618 (37.6)	486 (37.2)	132 (39.2)
	Male	974 (59.3)	797 (61.0)	177 (52.5)
	Nonbinary	25 (1.5)	11 (0.8)	14 (4.2)
	No response	26 (1.6)	12 (0.9)	14 (4.2)
**Race, n (%); *χ*^2^=41.21, *P*<.001**				
	Asian	55 (3.3)	42 (3.2)	13 (3.9)
	Black/African American	102 (6.2)	73 (5.6)	29 (8.6)
	White	1347 (82.0)	1107 (84.8)	240 (71.2)
	Other	139 (8.5)	84 (6.4)	55 (16.3)
**Ethnicity, n (%); *χ*^2^=3.71, *P*=.05**				
	Latinx	125 (7.6)	91 (7.0)	34 (10.1)
	Not Latinx	1518 (92.4)	1215 (93.0)	303 (89.9)
**Political ideology, n (%); *χ*^2^=103.31, *P*<.001**				
	Very conservative	90 (5.5)	50 (3.8)	40 (11.9)
	Conservative	268 (16.3)	182 (13.9)	86 (25.5)
	Moderate	613 (37.3)	517 (39.6)	96 (28.5)
	Liberal	316 (19.2)	290 (22.2)	26 (7.7)
	Very liberal	154 (9.4)	136 (10.4)	18 (5.3)
	No response	202 (12.3)	131 (10.0)	71 (21.1)

For the logistic regression analysis that tested factors associated with vaccine uptake and intent, we included those who reported their gender as male or female, reported their political ideology, and answered all theory-based vaccine items, resulting in a full case analysis (n=1370, 83%). We present results from the adjusted logistic regression models in Table 2. In the model with demographic covariates, only age, race, and political ideology were significantly associated with vaccine uptake/intent (all *P*<.01). Specifically, as age increased, the odds of being in the vaccinated/intenders group increased (adjusted odds ratio [AOR] 1.05, 95% CI 1.04-1.06). The “other” race category had lower odds of being in the vaccinated/intenders group than White participants (AOR 0.47, 95% CI 0.27-0.83). The odds of being in the vaccinated/intenders group increased across the political spectrum from a very conservative to a very liberal political ideology, such that those who reported being very liberal had more than 15 times the odds of being in the vaccinated/intenders group compared to those who reported being very conservative (AOR 15.77, 95% CI 7.03-35.35).

However, when theory-based attitudes and belief variables were added to the model, the only demographic variable that remained significant was age. Race and political ideology were no longer significant when controlling for attitudes and beliefs. The attitudes and beliefs variables associated with an increased odds of being in the vaccinated/intenders group included attitudes (AOR 3.72, 95% CI 2.42-5.73), injunctive norms (AOR 1.60, 95% CI 1.18-2.17), descriptive norms (AOR 1.59, 95% CI 1.14-2.22), self-efficacy (AOR 1.75, 95% CI 1.34-2.29), and perceived susceptibility to COVID-19 (AOR 1.30, 95% CI 1.04-1.64). Attitudes and beliefs associated with a decreased odds of being in the vaccinated/intenders group included a concern about side effects (AOR 0.59, 95% CI 0.46-0.76) and lack of trust in the vaccine (AOR 0.73, 95% CI 0.56-0.95). The only attitudes and beliefs variable that was not significantly associated with vaccine uptake/intent was a fear of needles.

**Table 2:** Results of logistic regression complete case analysis (N=1370).

Characteristics		Model 1: demographic covariates only, AOR^a^ (95% CI)	Model 2: demographic covariates plus theory-based factors, AOR (95% CI)
Age (years)		1.05^b^ (1.04-1.06)	1.03^c^ (1.01-1.05)
**Gender**			
	Female (reference)	N/A^d^	N/A
	Male	1.05 (0.77-1.45)	0.91 (0.52-1.59)
**Race**			
	White (reference)	N/A	N/A
	Asian	0.90 (0.37-2.22)	1.06 (0.23-4.88)
	Black/African American	0.77 (0.41-1.46)	1.15 (0.40-3.30)
	Other	0.47^c^ (0.27-0.83)	1.08 (0.40-2.94)
**Latinx ethnicity**			
	No (reference)	N/A	N/A
	Yes	1.20 (0.64-2.26)	1.32 (0.45-3.89)
**Political ideology**			
	Very conservative (reference)	N/A	N/A
	Conservative	1.75^e^ (1.03-2.96)	0.66 (0.22-1.95)
	Moderate	5.19^b^ (3.11-8.67)	0.85 (0.30-2.43)
	Liberal	13.80^b^ (7.20-26.43)	1.07 (0.33-3.54)
	Very liberal	15.77^b^ (7.03-35.35)	0.93 (0.22-3.92)
Attitudes		N/A	3.72^b^ (2.42-5.73)
Injunctive norms		N/A	1.60^c^ (1.18-2.17)
Descriptive norms		N/A	1.59^c^ (1.14-2.22)
Self-efficacy		N/A	1.75^b^ (1.34-2.29)
Susceptibility to COVID-19		N/A	1.30^e^ (1.04-1.64)
Side-effects barrier		N/A	0.59^b^ (0.46-0.76)
Fear-of-needles barrier		N/A	1.12 (0.91-1.36)
Do-not-trust-vaccine barrier		N/A	0.73^e^ (0.56-0.95)

^a^AOR: adjusted odds ratio.

^b^*P*<.001.

^c^*P*<.01.

^d^N/A: not applicable.

^e^*P*<.05.

## Discussion

### Principal Findings

This study examined hesitancy in COVID-19 vaccine uptake and intent using a national sample from the United States. Overall, vaccine uptake and intent were high in this sample, with almost 80% of the participants indicating they either received a COVID-19 vaccine already or intended to receive one when it was available to them. However, approximately 1 in 5 participants indicated they had not received the vaccine and did not report they definitely would receive it, when available, indicating vaccine hesitancy. With highly contagious viral variants quickly spreading across the nation, public health officials perceive a new phase of the ongoing COVID-19 pandemic being dubbed a “pandemic of the unvaccinated” [29]. There is an urgent need to understand the beliefs and attitudes associated with vaccine hesitancy so that interventions to improve the vaccination rate worldwide can be developed and implemented.

### Comparison With Prior Work

Our study found 3 demographic variables associated with vaccine uptake and intent in the model that included demographic characteristics only: age, race, and political affiliation. However, only age remained significant when accounting for the theory-based factors. Specifically, older age was associated with increased odds of being vaccinated or intending to be vaccinated. This is not surprising, given the vaccine rollout in the United States occurred largely by age group and is consistent with early research prior to vaccine availability that noted increasing age was associated with increasing vaccine intent [12]. All adults in the United States were eligible for vaccination by April 19, 2021 [30]. It is possible some of the adults who responded were not eligible for vaccination yet, because these data were collected in February and March. However, because we included people who reported they definitely would get the vaccine when it was available in with the vaccinated sample, this should not have affected our results. The association between age and vaccine uptake and intent may be due to the fact that older adults, if infected, are more likely to have severe disease [31]. However, this association persisted even when controlling for perceived susceptibility to COVID-19, indicating the association may not be explained by either availability or perceived susceptibility. Our study did not examine issues of access or logistics, particularly transportation barriers, time off work, and childcare, which likely affect younger adults more than older adults. Access and logistical barriers are important issues to examine in future research.

Political affiliation was also associated with vaccine uptake and intent, with the odds of uptake increasing across the sample, with very conservatives reporting the lowest uptake/intent and very liberals reporting the highest uptake/intent. However, this association was no longer significant when accounting for attitudes and beliefs. Another recent research study found increased vaccine hesitancy among moderates and conservatives (compared to liberals) when accounting for respondent characteristics and behaviors [32]. However, this research did not include beliefs in the model, which our data indicate may be an important predictor to analyze. An additional study examined COVID-19 vaccine intent while controlling for political affiliation and media exposure [33]. This study did find a difference in intent between Republicans and Democrats, with Democrats indicating a higher intent to be vaccinated. Although they controlled for preferred media for virus-related news (including social media Fox News, and CNN/MSNBC), and belief in conspiracy theories, they did not control for other attitudinal or belief variables, including injunctive and descriptive norms. It is essential to understand that this lack of association once we control for attitudes and beliefs does not imply political affiliation’s lack of causal effect on vaccine hesitancy. Other political science research has found that instead of people’s moral foundations predicting their political affiliations, it is in fact people’s political affiliations that predict their moral foundations [34]. That is, people tend to switch their moral values, depending on how they fit with their political beliefs, as opposed to switching their political beliefs, depending on how they fit their moral values. Based on these findings, it is important for future research to examine the interplay between political affiliation, attitudes, and beliefs to better understand which is actually the driver of the association with vaccine hesitancy. Having a better understanding of the association between political affiliation, attitudes, beliefs, and vaccine hesitancy will enable researchers to develop community-based interventions that address these challenges.

Like political affiliation, race was significantly associated with uptake/intent in the model that included demographic characteristics only. Specifically, people who reported they were a race other than White, Asian, or Black/African American were approximately half as likely to be vaccinated or intend to be vaccinated compared to the White participants. However, this association was no longer significant when theoretical covariates were entered into the model. As was discussed earlier in regard to age, our study did not examine issues of access or logistics, particularly transportation barriers, time off work, and childcare, which may affect non-White respondents more than White respondents. Although research does indicate there is mistrust among non-White patients, there are also issues with health equity and access to care that seem to be driving the disparity [35]. A recent publication noted that the racial disparity in COVID-19 mortality is due more to structural racism than to race itself [36]. It is also important to note that although the association we found in our study was significant for the “other” race category, it was not significant for Black/African American participants or those who reported Latinx ethnicity. Future research should examine these associations to better understand the interplay between race, attitudes, beliefs, and vaccine hesitancy so that culturally appropriate community-based interventions can be developed.

The primary aim of this study was to identify the beliefs underlying the US adults’ decision to get vaccinated against COVID-19. Of note, when we added the theory-based constructs to the regression model for vaccine uptake and intention, age remained the only statistically significant demographic variable. This points to the important contributions of the theoretical constructs in explaining the variation in the decision to get vaccinated, beyond the influence of several demographic factors. The theoretical construct most strongly associated with vaccine uptake and intention in this sample was attitude. This finding suggests that attitude could be an important focal point for interventions aimed at increasing COVID-19 vaccine uptake. Attitudes can be addressed through communication and education campaigns that present the advantages of getting vaccinated and address any potential negative consequences. One method some hospital systems have used is publishing infographics that demonstrate that the hospitalized patients are overwhelmingly unvaccinated [37]. Furthermore, a multilevel intervention that included a component addressing patient and provider attitudes toward human papillomavirus vaccination saw increased uptake of the vaccine in the intervention group compared to the control group [38]. However, the authors stated the increase was lower than expected. Future research should examine effective ways to improve attitudes and increase uptake of vaccines.

Self-efficacy was also significantly associated with vaccine uptake and intention in this sample. This suggests that public health interventions should address adults’ confidence that they can get vaccinated. There are 2 approaches to improving self-efficacy or capacity. One approach aims to address people’s beliefs directly. Communication and educational campaigns can potentially help people see and come to believe that they have the capacity to get vaccinated. Modeling is 1 effective way to improve self-efficacy [39]. According to past research, modeling interventions should resemble the target group, start with small steps, look to succeed but not immediately, and be reinforced for the behavior of getting vaccinated [40]. Thus, these campaigns could include examples of how people successfully overcame their hesitancy to get the vaccine. The second approach is to address the actual environment by removing barriers to getting vaccinated or adding facilitators at local, organizational, and governmental levels. This could include removing the request for health insurance information and providing paid time off from work to get the vaccine and recover from any short-term side effects.

Both types of normative beliefs (injunctive and descriptive) were significantly associated with vaccine uptake and intention, albeit less strongly so than attitude and self-efficacy. Injunctive norms represent people’s perceptions about what people who are important to them think they should do, and descriptive norms represent people’s beliefs about how people like them are behaving. This suggests that, in this sample of US adults, the influence of important people in their lives and people like them might be key determinants of their intention to get vaccinated. Therefore, health communication messages tailored for US adults should emphasize that people important to them want them to get vaccinated and people like them are getting vaccinated.

Two of the barriers examined were associated with decreased odds of being in the vaccinated/intenders group. Specifically agreeing that the vaccine would cause side effects that would interfere with their usual activities and reporting they do not trust the vaccine were both associated with decreased odds of being vaccinated/intending to get vaccinated. This is consistent with other recent surveys examining people who have not yet been vaccinated and found that almost 1 in 5 of them reported not being vaccinated due to concerns over adverse effects or the vaccines’ newness [41]. Many of these concerns among the population stem from misinformation encountered on social media. Indeed, 1 recent research study found that COVID-19 vaccine intent is significantly associated with not relying on social media for virus information [33]. Misinformation can shape people’s decision-making and perceptions, particularly if left unchallenged [41]. Specifically, 1 study found that negative television news coverage of a medication can increase reporting of adverse events for that medication [42]. Furthermore, research shows that viewing a website critical of vaccines for just 5-10 minutes decreases the intention to vaccinate [43]. However, it is important to be transparent about the potential side effects of any medication or vaccine. Research in the HIV literature found that a failure to acknowledge potential negative effects of receiving an HIV test results in a “boomerang effect,” where people who already perceive obstacles to testing are less likely to get tested if the negative effects aren’t acknowledged [44]. However, to foster trust in these vaccines and combat the misinformation that people encounter regarding safety and efficacy, it is important to challenge their misperceptions and provide scientifically accurate information that is understandable to the layperson and delivered by a person they trust. This information should include that the vaccine side effects are mild, the risks of the vaccine are much lower than the risks of COVID-19 infection, and the vaccines are effective in preventing severe COVID-19. A key partner in this conversation is the person’s health care provider, and providers should communicate to their patients that they strongly recommend vaccination. Research shows the intent to be vaccinated increases if the person’s health care provider recommends they receive the vaccine [12].

### Limitations

Although this study had numerous strengths, including using a national sample and examining relevant and understudied attitudes and beliefs, the results should be interpreted in the light of some limitations. First, these data are cross-sectional and causal associations cannot be determined. Second, the data were collected in February and March 2021. It is possible attitudes, intent, and uptake may have shifted in the intervening months. This period was slightly before all US adults could be vaccinated against COVID-19 and was also prior to widespread infection with the more contagious delta and subsequent omicron variants. Ongoing research on these topics is warranted. Third, although we did recruit nationally for this study, compared to the overall US population, our sample was a lower proportion of females (37.6% vs 50.8% nationwide) and Hispanic (7.6% vs 18.5% nationwide) and was older (mean age 59.4 years vs median age 37.7 years nationwide) [45]. Although we controlled for demographic variables in the regression analyses, our findings may not be generalizable to the broader US population. In addition, our recruitment strategy using Microsoft News limited our sample to only those who use Microsoft products and have this feature turned on, further limiting generalizability.

### Conclusion

Vaccination represents one of the best tools to combat the ongoing COVID-19 pandemic [46]. Hesitancy regarding vaccines persists among adults in the United States, despite overwhelming scientific evidence of safety and efficacy. These beliefs result in lower uptake of vaccines and unnecessary morbidity and mortality from vaccine-preventable diseases. This research provides novel insight into the association between attitudes and beliefs with vaccine hesitancy. Specifically, older age, attitudes, injunctive norms, descriptive norms, and self-efficacy are positively associated with vaccine uptake and intent, whereas perceived side effects and lack of trust in the vaccine are associated with lower uptake and intent. Before vaccine hesitancy can be addressed, researchers need to understand the basis of vaccine hesitancy and intent as well as which populations may show higher hesitancy to the COVID-19 vaccine so that interventions can be adequately targeted.

## Abbreviations

AOR: adjusted odds ratio
FDA: Food and Drug Administration
HBM: Health Belief Model
RAA: reasoned action approach
